# A Review of Intra- and Extracellular Antigen Delivery Systems for Virus Vaccines of Finfish

**DOI:** 10.1155/2015/960859

**Published:** 2015-05-03

**Authors:** Hetron Mweemba Munang'andu, Øystein Evensen

**Affiliations:** Section of Aquatic Medicine and Nutrition, Department of Basic Sciences and Aquatic Medicine, Faculty of Veterinary Medicine and Biosciences, Norwegian University of Life Sciences, Ullevalsveien 72, P.O. Box 8146, 0033 Oslo, Norway

## Abstract

Vaccine efficacy in aquaculture has for a long time depended on evaluating relative percent survival and antibody responses after vaccination. However, current advances in vaccine immunology show that the route in which antigens are delivered into cells is deterministic of the type of adaptive immune response evoked by vaccination. Antigens delivered by the intracellular route induce MHC-I restricted CD8+ responses while antigens presented through the extracellular route activate MHC-II restricted CD4+ responses implying that the route of antigen delivery is a conduit to induction of B- or T-cell immune responses. In finfish, different antigen delivery systems have been explored that include live, DNA, inactivated whole virus, fusion protein, virus-like particles, and subunit vaccines although mechanisms linking these delivery systems to protective immunity have not been studied in detail. Hence, in this review we provide a synopsis of different strategies used to administer viral antigens via the intra- or extracellular compartments. Further, we highlight the differences in immune responses induced by antigens processed by the endogenous route compared to exogenously processed antigens. Overall, we anticipate that the synopsis put together in this review will shed insights into limitations and successes of the current vaccination strategies used in finfish vaccinology.

## 1. Introduction

The central hallmark of vaccination is to prime the adaptive immune system to develop immune responses that will protect the host organism upon a second encounter with the same pathogen. However, priming the adaptive immune system requires activation of naïve B- and T-lymphocytes into effector cells that translate into protective immunity. While studies on the immunological basis of vaccine protection have for a long time focused on humoral and cellular responses as measures of protective immunity, growing evidence shows that the mode by which antigens are presented to B- or T-lymphocytes has a significant influence on the outcome of adaptive immune responses induced by vaccination which is also influenced by the mode in which antigens are administered to host cells [1, 2]. Put together, these elements drive vaccine development into a cross-talk between vaccinology and immunology in which vaccine design and its delivery (vaccinology) on one hand have to be optimized in order to gain an effective immune response (immunology) on the other. Hence, optimization of antigen design and its delivery into host cells is a prerequisite to inducing an optimal protective immune response.

Unlike B-lymphocytes, which are precursors of antibody secreting cells that can recognize antigens through primed antigen presenting cells (APCs)/activated B-cells [1], T-cell receptors (TCRs) can only “see” antigens that are processed and presented by APCs. TCRs recognize antigen peptides bound on the surface of MHC molecules [2]. Endogenous peptides derived from intracellular sources such as replicating virus are synthesized and processed for presentation to naïve CD8 T-cells by MHC-I molecules while exogenous peptides derived from extracellular sources are processed and presented to naïve CD4 T-cells by MHC-II molecules. An alternative mechanism that permits some extracellular antigens to activate naïve CD8 T-cells called cross presentation exists which occurs via the MHC-I pathway [3, 4]. For antigens delivered via the endosomal route, proteosomes degrade soluble antigens after ubiquitination which have been synthesized in the cytosol or escaped to the endoplasmic reticulum (ER) by cross presentation [5]. Thereafter, the processed antigens are released after proteosomal degradation to generate peptides that are transported into the ER by the transporter-associated antigen processing (TAPs) [5, 6]. Once in the ER, the antigenic peptides are loaded onto MHC-I molecules for presentation on the cell surface where they initiate the activation of naïve CD8 T-cells into effector cytotoxic T-lymphocytes (CTLs) [7–9]. In the case of antigens delivered by the exogenous route, lysosomes degrade endocytosed antigens after endosomal fusion with lysosomes [10]. In general, lysosomes can degrade complex structures such as whole viral particles that are delivered to them via endocytosis by the extracellular route [11]. Presentation of processed peptides by endosomal degradation leads to maturation of APCs into professional APCs which is characterized by expressing MHC-II molecules and antigen specific signaling molecules such CD40L, CD80, and CD86. The resulting professional APCs are the prime initiators of adaptive immune responses that activate naïve T-cells into effector cells through the MHC-peptide complexes and immune modulation molecules. Therefore, it follows that, for a vaccine antigen to turn naïve B- or T-lymphocytes into “protective” cell, there has to be an efficient antigen delivery system that stimulates the activation of cell of the adaptive immune system.

Although studies on antigen presentation in fish immunology have gained prominence in recent years [12–14], there is still limited research on activation of cells of the adaptive immune system by APCs, which precludes our understanding of the role of innate immunity in optimizing vaccine performance. Despite that, several studies have been carried out trying to deliver viral antigens into different compartments of fish cells. Hence, in this review we provide an overview of these delivery systems and based on this approach we highlight the different immune responses induced by antigens delivered by the intracellular route compared to antigens delivered by the extracellular route. In addition, we also highlight the differences in vaccine efficacy from fish immunized using antigens delivered by the exogenous route compared to fish vaccinated using the endogenous route. Overall, we anticipate that the synopsis of different antigen delivery systems put together in this review will shed new insights into limitations and successes of the current vaccination strategies used in fish vaccinology.

## 2. Fish Antigen Presenting Cells, Adaptive Immune Cells, and Their Receptors

In mammals, antigen presentation is carried out by different cell types that include monocytes, macrophages, and dendritic cells [15]. These cells possess pattern recognition receptors (PRRs) that recognize and bind to pathogen associated molecular patterns (PAMPs) known as “danger signals” on pathogens [16]. Upon binding to PAMPs using PRRs, monocytes mature into macrophages while immature dendritic cells (DCs) also transform into mature dendritic ones to become professional APCs, which induce the expression of proinflammatory cytokines that attract more APCs to the sites of antigen deposition [15, 17]. Upon encounter with the APCs, naïve B- and T-cells undergo maturation to become memory cells capable of recognizing the antigens in subsequent encounters thereby creating the basis acquired immunity.

In teleosts fish, APCs known to possess PRRs having the capacity to bind to different PAMPs on pathogens have been described in different species and these include monocytes, macrophages, and dendritic-like cells [13, 18–26]. In addition fish B-cells have been shown to carry out antigen presentation apart from their role as antibody secreting cells [27]. As a result,* in vitro* methods for culturing fish monocytes, macrophages, and dendritic-like cells have been developed which makes it easy to study antigen presentation using cell cultures [18, 19, 24, 25]. It is interesting to note that TAP genes comparable to those seen in mammals have been identified and mapped to MHC regions in different cartilaginous and bony fish species suggesting that similar mechanisms of endogenous antigen processing seen in higher vertebrates also exist in teleosts fish [28–31]. Unlike in mammals where APCs carrying processed antigens migrate to the lymph nodes [32], in fish APCs carrying antigens migrate to the head, kidney, and spleen [13], which are the major lymphoid organs [33]. Apart from lymphoid organs, APCs have been detected in other organs such as the gills, skin, and intestines in fish [34, 35]. In addition, different phagocytic cell types have been characterized in fish although their antigen presentation capabilities have not been investigated [36, 37].

Similar to their mammalian counterparts, fish APCs possess a wide range of surface markers that include CD80/CD86, CD83, CD209, MHC-I, and MHC-II proteins [20, 38–41]. In fish, CD83 has been shown to be an activation marker for macrophages [42] and dendritic-like cells [23]. Apart from CD83, other surface markers identified for fish dendritic-like cells include CD208/lysosomal associated membrane protein (LAMP3) [43]. Recently, Zhu et al. [44] showed that fish B-cells act as pivotal APCs in priming the adaptive immune system using CD80/CD86 molecules. In another study, Abós et al. [45] showed upregulation of MHC-II genes that coincided with upregulation of CD80/CD86 genes in a nonlethal infection (no cytopathic effects observed) of viral hemorrhagic septicemia virus (VHSV) in IgM+ cells which consolidates the notion that fish B-cells use CD80/CD86 molecules to activate the adaptive immune system using the MHC-II pathway [44, 46].

As shown in Table 1, all four T-cell receptor chains (*α*, *β*, *γ*, and *δ*) required for binding to APCs together with the four chains (*γ*-, *δ*-, *ε*-, and *ζ*-chain) of the CD3 coreceptor complex required for T-cell activation in mammals have been reported in fish. In addition, the T-cell costimulatory marker CD28 and the negative regulatory marker CTLA-4, which bind to CD80 and CD86 receptors on APCs, have also been characterized in fish [47, 48]. As for CD8 T-cells, two subsets have been characterized, namely, CD8*α* and CD8*β*, from different fish species of which CD8*α* has been the most widely used marker for T-cell activation in different studies [49–51]. Moreover, cell mediated cytotoxicity against allogeneic targets and virus infected cells has been reported by different scientists [50, 52–54]. Put together these observations suggest that fish T-cells possess surface receptors essential for the binding to APCs comparable to those found in mammals and that activation of T-cells into effector cytotoxic T-lymphocytes (CTLs) could be based on similar mechanisms to those seen in mammals.

There are three immunoglobulin isotypes characterized in fish, this far, and these include IgM [55, 56] and IgD [55, 57] also present in mammals while the recently identified IgT [58] is only found in fish where it exists as membrane bound and secreted form in serum [59, 60]. IgM is the most abundant isotype in serum where it is estimated to be >1000-fold higher than IgT [61, 62]. In addition, IgM has been detected in the mucus of the skin, gills, and intestines although its levels in these organs are far much lower than levels detected in serum [61, 62]. On the contrary, IgT is predominantly found in the mucus of the skin, gut, and intestines [61–63] where it is >100-fold higher than levels detected in sera [61]. It is interesting to note that the costimulatory marker CD40L mostly expressed in activated CD4+ T-cells, which binds to the CD40 receptors on APCs in order to activate B-cell proliferation [64], has been characterized in fish [65]. In addition, transcription factors involved in specification of CD4 T-cells into different T-helper (Th) subtypes have also been characterized, which include T-bet [66, 67], GATA-3 [68–70], and ROR*γ* [71, 72] for the differentiation of naïve CD4 T-cells into Th1, Th2, and Th17 subtypes, respectively. In addition, several cytokines linked to specification of CD4 T-cell into different subtypes have been characterized and these include IL-2, IL-4, IL-6, IL-10, IL-12, IFN*γ*, IL-15, IL-21, IL-22, and TGF*β* [40, 41, 73, 74]. Overall, the characterization of different APCs and adaptive immune cells together with their receptors and regulatory cytokine presented here suggests that teleosts fish antigen presentation mechanisms could be comparable to those used by mammals suggesting that antigen presentation mechanisms have been conserved across the vertebrate taxa.

## 3. Intracellular Antigen Delivery Systems 

**Figure 1:**
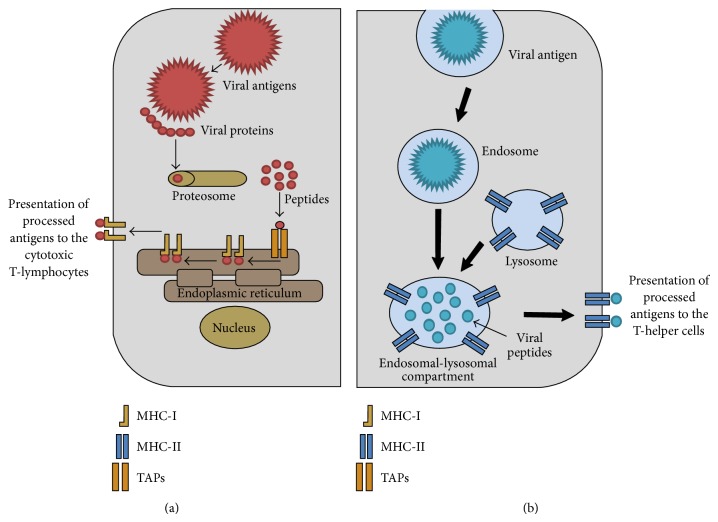
The endogenous and exogenous pathways of viral antigen entry into host cells. (a) Endogenous pathway shows viral antigens that enter the host cells by the intracellular route. Once internalized, the viral antigens are degraded into peptides by proteasomes. Thereafter, the processed antigenic peptides are transported via the transporter associated with antigen presentation (TAPs) to the endoplasmic reticulum (ER) where they are loaded onto MHC-I molecules for presentation at the cell surface to CD8+ T-cells. (b) Exogenous pathway shows antigens that enter the antigen presenting cells (APCs) via the extracellular route which results in internalization of the antigens in the endosomes. Thereafter, the endosomes fuse with the lysosomes to form the endosomal-lysosomal compartments that have MHC-II complexes. In the endosomal-lysosomal compartments, the antigens are degraded into peptides followed by packaging of the peptides onto MHC-II complexes. Thereafter, the MHC-II complexes carrying the peptides are transported to the cell surface for presentation of the antigenic peptides to the CD4 T-cells.

Intracellular antigen delivery systems involve immunization strategies that administer the vaccine antigens into the cytoplasm (Figure 1) and these include the following.

### 3.1. Live Vaccines

Live vaccines use attenuated viruses or recombinant antigens encoded by live virus vectors that have the capacity to replicate in host cells with attenuated pathogenicity lacking the ability to cause disease. As analogues of pathogenic viruses, they engage with the cell membrane by binding to surface receptors using epitopes similar to their native virus, thereby gaining entry into endosomal structures and the cytosol where they use the host cell machinery to replicate. Consequently, the processed antigens are presented on the cell surface by MHC-I molecules while soluble antigens expressed by replicating virus are engulfed by APCs to induce humoral immune responses (Figure 1). Hence, live vaccines induce both cellular and humoral immune responses.

In general, different scientists have reported the induction of CTL responses in fish [52, 53, 75]. Utke et al. [76] showed activation of the CTLs by viral hemorrhagic septicemia virus (VHSV) infection in rainbow trout, while we [51] recently showed activation of eomesodermin, a transcription factor involved in activation of CD8*α* cells in Atlantic salmon exposed to infectious pancreatic necrosis virus (IPNV). Similarly, Chang et al. [49] showed activation of CD8*α* cells after exposing orange spotted grouper (*Epinephelus coioides*) to nervous necrosis virus (NNV). Flow cytometry analysis of the spleen cells from fish exposed to NNV showed increased mean fluorescent intensity of the CD8*α* cells and peripheral blood leukocytes (PBLs) which were linked to increased cytotoxicity and MHC-I restriction of the sorted lymphocytes by recombinant CD8*α* antibodies. Several fish species have shown upregulation of MHC-I and -II molecules [49, 77–80] as well as expression of high antibody levels after exposure to viral infections [81, 82] suggesting that attenuated viruses administered as live vaccines could evoke both cellular and humoral immunity. Based on these observations, several attempts have been made to develop live viral vaccines for fish (Table 2) and some of the strategies explored this far are outlined below.

#### 3.1.1. Natural Selection of Avirulent Strains

Roberti et al. [83] discovered a naturally attenuated mutant of infectious hematopoietic necrosis virus (IHNV) that conferred protection against IHNV in rainbow trout although the vaccine resulted in causing low level mortality after challenge. Adelmann et al. [84] used an oral vaccine against VHSV obtained from a naturally attenuated live virus selected using monoclonal antibodies. In their study, they [84] showed high expression levels of MHC-II and CD4 mRNAs. In addition, they detected antibody responses that were linked to significant protection in rainbow trout after challenge.

#### 3.1.2. Attenuation by Serial Passages

An attenuated IHNV vaccine was developed at Oregon State University by multiple passages using a rainbow trout isolate propagated using the steelhead trout cell culture [85, 86]. The vaccine showed high protection (95%) in vaccinated Chinook salmon while mortality in control fish reached 90%. Although the vaccine was highly protective in Chinook salmon, when used in rainbow trout it showed significant mortality and as such it was stopped [87, 88]. Since the Ab strain of infectious pancreatic necrosis virus (IPNV) was found to be less virulent than the West Buxton, Sp, or Jasper strain, Dorson et al. [89] attempted to develop an attenuated strain of IPNV from the Ab strain after several passages on RTG cells. Neither the serially passaged nor the original Ab strain conferred protection.

#### 3.1.3. Reverse Genetics

Reverse genetics has been used in fish vaccinology to generate avirulent strains for use as live vaccines. For example, recombinant IHNV having a deletion of the NV gene resulted in irreversible attenuation of the wild type virulent strain resulting in the induction of high protection levels in rainbow trout [90]. In another study, recombinant IHNV generated by replacing the NV gene with green fluorescent protein (GFP) or substituting the IHNV G-gene with the G-gene of VHSV induced heterologous protection in rainbow trout [91]. For IPNV, reverse genetics was used to generate an avirulent strain for use as a live vaccine against the wild type strain by substituting amino acids on positions 217 and 221 of the VP2 capsid [92]. These studies showed that the strain encoding the T_217_A_221_ motif caused high mortality in Atlantic salmon while the strain encoding the P_217_T_221_ motif was avirulent and linked to subclinical infections [93]. Infecting Atlantic salmon with high and low virulent strains at a nonpermissive physiological state (presmoltification stage) did not result in mortality. When the vaccinated fish were challenged at smolt stage (permissive) the avirulent strain was less immunogenic than the virulent vaccine strain [82]. In addition, we observed that the avirulent strain reverted to virulence under stress conditions [37, 94]. In general, the fear of reversion to virulence has been the major hindrance for the licensure of live vaccines in aquaculture.

### 3.2. DNA Vaccines

The strategy of DNA vaccination is based on the principle that the encoded immunogenic protein is injected into the muscle or other tissues where it enters the host cells and directs the synthesis of its polypeptide antigen from the plasmid vector. Once transfected into host cells, transcribed antigens replicate in the cytosol using the endogenous pathway while soluble or secreted antigens are phagocytized by APC and gain access into the exogenous pathway (Figure 1). In principle, DNA vaccines result in the* in vivo* synthesis of antigenic proteins using the host cell machinery in a manner identical to natural virus infection in the case of DNA vaccines made for viral diseases. This culminates into antigenic proteins expressed by plasmid DNA gaining access to both the exogenous and endogenous pathways in the activation of both humoral and cellular mediated immune responses.

Boudinot et al. [95] demonstrated the intracellular delivery of the plasmid DNA encoding the recombinant G protein of VHSV inside the muscle cells of vaccinated rainbow trout. Intracellular detection of the G-protein was shown up to 45 days at the injection sites. Transcription of the G-protein was demonstrated by detection of mRNA in muscle tissue extracts, which was linked to expression of high antibody and MHC-II mRNAs levels. In another study, Utke et al. [96] showed activation of the CTLs following immunization using the G-protein of VHSV in rainbow trout. In their study, they [96] used PBLs collected from fish immunized with a DNA vaccine encoding the recombinant G -protein of VHSV and showed that PBLs from vaccinated fish killed the VHSV MHC-I matched RTG-2 cells indicating that the G-proteins had the capacity to induce CTL responses in vaccinated fish. They also showed the homing of leukocytes to the injection site suggesting that cells expressing the recombinant G-protein had a chemoattractant effect. This observation was recently supported by Castro et al. [97] who showed that B-lymphocytes, both IgM^+^ and IgT^+^ cells, represent one of the major cell types infiltrating the injection sites expressing the G-protein of VHSV. In their study, they showed upregulation of CXCR3B, a receptor for CXCL11, together with CK5B and CK6 chemokines, which could play chemotactic roles in the early recruitment of B-cells at the injection sites. Put together, these studies show that the intracellular expression of proteins transcribed from DNA vaccines in fish cells leads to homing of leukocytes and B-cells to injection sites with possible involvements of chemoattractant chemokines. Further, these studies suggest that antigens delivered by this endogenous route evoke both the humoral and cellular mediated immune responses in vaccinated fish.

Finally, it is important to point out that immunization using DNA vaccines exhibits many advantages over the live and inactivated vaccines. Intracellular synthesis of the antigenic proteins poses no danger of reversion to virulence and does not require inactivation of viruses using toxic substances. High expression levels of humoral and cellular responses can be achieved at low doses as shown by Corbeil et al. [98] that nanogram quantities of a DNA vaccine protected rainbow trout against IHNV infection after challenge. In addition, intracellular synthesized antigens tend to fold in their native conformation and correctly glycosylated displaying the neutralizing epitopes in a similar pattern to the native virus [99]. In terms of genetic engineering, combinational approaches can easily be adopted. For example, the use of molecularly encoded cytokine adjuvants like IL-2 in DNA engineered vaccines has shown the ability to enhance DNA delivery and increase the duration and magnitude of plasmid DNA expression* in vivo* [100]. Jimenez et al. [101] coinjected recombinant IL-8 with plasmid DNA encoding the G-protein of VHSV in rainbow trout and showed massive infiltration of neutrophils at the injection site linked to upregulation of proinflammatory cytokines.  Table 3 shows the DNA vaccines explored for use in fish, this far, of which only the DNA vaccine for IHNV has been licensed in Canada (Novartis Ltd.).

### 3.3. Fusion Protein Vaccines

**Figure 2:**
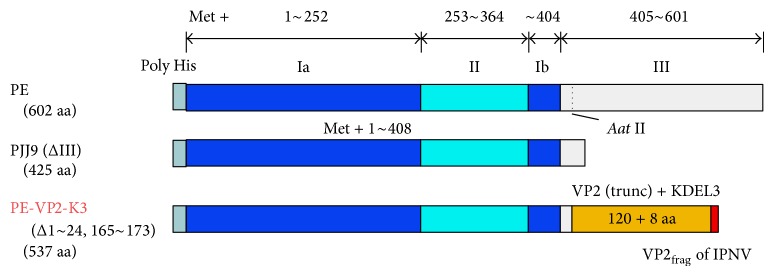
The fusion of the VP2- of IPNV to the* Pseudomonas aeruginosa *exotoxin A (PE). PE shows a 602 aa exotoxin for* Pseudomonas aeruginosa* made of three domains. Domain Ia (blue) located on 1–252 aa followed by domain II (green) on the location 253–364 aa, domain Ib (blue) located on 365–404 aa, and finally domain III on the extreme end located on 405–601 aa. PJJ9 (ΔIII) is a 425 aa long intermediate phase in which the toxic moiety of domain III has been cleaved. PE-VP2-KE is the final construct in which the toxic moiety of domain III has been replaced with the truncated VP2 (yellow) immunogenic protein of infectious pancreatic necrosis virus (IPNV). Note that the VP2 is attached to the KDEL3 signaling peptide (red).

This mode of antigen delivery which has been referred to as “the first class ticket to induction of MHC-I responses” relies on receptor mediated internalization of viral antigens to the ER followed by retrograde translocation into the cytosol [102]. Only a few studies have explored this delivery system in fish vaccinology, this far [103, 104]. Li-Li et al. [104] constructed a fusion protein vaccine made by fusing the VP2-VP3 polyprotein of IPNV with the exotoxin of* Lactobacillus casei*, which resulted in reduced viral loads in vaccinated rainbow trout after challenge while in our group we constructed a fusion protein vaccine made by fusing the VP2 of IPNV with the* Pseudomonas aeruginosa* exotoxin A (EP) [103]. The exact mechanisms in which viral antigens are translocated into the cytosol are dependent on three bacterial proteins as illustrated from the PE fusion protein in Figure 2. The PE protein has three functional domains, namely, the receptor binding domain-I, transmembrane targeting domain-II, and the toxic moiety domain-III. Domain-I binds to the *α*2-macroglobulin receptor on the cell surface. After binding to domain-I the ligand-receptor complex is internalized through the receptor mediated endocytosis. After enzymatic cleavage by the protease furin in the endosome, the protein fragment encoding domains-II and -III is delivered into the Golgi by the ER retrograde transport and further into the cytosol using domain-II, which is responsible for transmembrane translocation of the toxin proteins into the cytoplasm. As shown in Figure 2, domain-III, which is toxic to cells, is eliminated and is replaced with the immunogenic protein (VP2) of IPNV bound to the KDEL signaling peptide. The purpose of including the KDEL signaling peptide in the final construct (PE-VP2-KDEL) is that it enables the binding of the whole construct to the Golgi membrane KDEL-receptor. Once bound to the Golgi membrane receptor, the ligand-receptor complex is packaged into vesicles for retrograde transport back to the ER where processed peptides are packaged on MHC-I molecules for presentation on the cell surface. In our studies, we employed the PE-VP2-KDEL fusion protein to deliver the VP2 immunogenic protein of IPNV intracellularly as a vaccine. Although we did not assess the CTL responses induced by this antigen delivery system, our findings show that these vaccines were able to induce a low level antibody response suggesting that antigens delivered using this method could gain access to induction of humoral responses in vaccinated fish. However, there is a need for detailed investigation to determine the role of CTL responses induced by this mode of antigen delivery in vaccinated fish.

### 3.4. Nanoparticle Vaccines

Polymeric nanoparticles formulated from biodegradable polymers have been widely explored as carriers for controlled delivery of vaccine antigens [105, 106]. This system can potentially deliver antigens to the desired location at predetermined rates and durations to generate an optimal immune response [107]. For example, Tian et al. [108–110] and Zheng et al. [111] showed that lymphocytic disease virus (LCDV) encapsulated in particles sustained a much longer release of the DNA antigen than naked DNA injected in Japanese flounder. In addition, carriers protect the antigen from degradation until release as shown by Rajeshkumar et al. [112] that encapsulated DNA antigens were protected from degradation by DNAase for vaccines used in the Asian sea bass (*Lates calcarifer*).

To deliver the antigens into host cells, nanoparticle materials are internalized by endocytosis [113]. To deliver the antigens into the cytosol, the release of antigens from the acidic endosomes requires membrane disruptive agents, which release the internalized proteins into the cytosol. Therefore, encapsulation carriers should include membrane penetrating peptides and polymers that disrupt the membranes when the pH declines in the endosomes. For example, Standley et al. [114] made acid degradable nanoparticles, which were designed to release encapsulated proteins in a pH-dependent manner. In their study, they made nanoparticles that were stable at pH 7.4 but quickly degradable at pH 5.0 in the acidic endosomal environment enabling the release of antigens into the cytosol, ultimately resulting in upregulation of MHC-I. Another method explored is the use of amphiphilic polymers [115–117], which also have pH-dependent membrane disruptive properties protonated at the endosomal pH range [115, 118]. Upon reduction of the endosomal pH, these particles increase their hydrophobicity to facilitate the disruption and penetration of the endosomal membranes culminating in the release of antigens in the cytosol. These amphiphilic polymers have been shown to increase CD8+ responses and to improve vaccine potency [119]. In summary, these studies show that nanoparticle antigen delivery systems can be designed to deliver antigens through the intra- or extracellular routes to evoke immune responses linked to the MHC-I or -II pathways.

Studies in higher vertebrates have shown that APCs easily carry out phagocytosis of nanoparticles and microparticles between 150 nm and 4.5 *μ*m [120, 121] with the optimal size for phagocytosis being 500 nm [122] while monocytes have been shown to easily phagocytose nanoparticles >100 nm [123]. And, as pointed out by Gutierro et al. [124], nanoparticles that encapsulate antigens resemble pathogens in terms of their uptake into host cells by mirroring the route of pathogen uptake and the immune response triggered after nanoparticle uptake. Fehr et al. [125] and He et al. [120] have also pointed out that nanoparticles can also be used to carry antigens on their surface which would serve as a good stimulant for the induction of B-cell responses. Although antigen delivery using nanoparticle vaccines has been well studied in higher vertebrates, indications are that fish cells use similar mechanisms of antigen uptake from nanoparticle based vaccines. For example, Ruyra et al. [126] showed entry of liposome-based nanoparticles in zebrafish hepatocytes and trout macrophages by endocytosis. Upon entry, the nanoparticle laden cells initiated specific proinflammatory responses while Fredriksen and Grip [127] showed intracellular cytoplasmic localization of polylactic-coglycolic acid (PLGA) nanoparticles in TO-cells [24]. These findings support earlier observations, which showed that because PLGA particles are less hydrophilic than alginates, they are easily incorporated into host cells, which makes them suitable vehicles for delivering antigens into intracellular compartments [128–131]. Recently, we [103] used PLGA nanoparticles to deliver inactivated whole viral particles of IPNV as a vaccine, which expressed low antibody levels comparable to those induced by inactivated whole virus (IWV) vaccines suggesting that delivery of antigens using PLGA nanoparticles has the ability to induce humoral immune responses in vaccinated fish. Although there are limited studies that categorically demonstrate the intracellular delivery of nanoparticle based vaccines in fish cells, Rajeshkumar et al. [112] were able to induce low level cytotoxicity (tested* in vitro*) using chitosan nanoparticle vaccines in Asian sea bass vaccinated against vibriosis (*Listonella anguillarum*).  Table 4 shows that only a few studies have been carried out using nanoparticle based technologies to administer viral antigens in fish. In general, indications show that nanoparticle based vaccines have the potential to deliver antigens into different host cell compartments and thus that they can induce cellular and humoral immune responses in vaccinated fish. The efficacy of nanoparticle vaccines needs to be improved and explored in more detail.

## 4. Extracellular Antigen Delivery Systems

This approach involves antigen delivery systems that administer viral antigens into the extracellular compartments using the exogenous pathway (Figure 1).

### 4.1. Inactivated Whole Viral Vaccines

This mode of antigen delivery ensures that the antigenic protein is preserved in its native structure while the virus is rendered nonreplicative using chemical or physical methods. Given that inactivated whole virus (IWV) vaccines are nonreplicative, it follows that their antigens enter the host cells by the exogenous route and their processed peptides are presented to CD4 cells via the MHC-II pathway. And, as such, several studies have shown upregulation of MHC-II genes in response to vaccination using IWV vaccines [77, 132]. In terms of CD4 T-cell differentiation, IWV vaccines have been shown to predominantly activate genes linked to the T-helper 2 (Th2) responses [133]. For example, we showed upregulation of GATA-3, a transcription factor linked to activation of naïve CD4 cells into Th2 responses, when genes linked to activation of Th1 and CD8 T-cell responses were downregulated [51]. In this study, we showed a high correlation between GATA-3 and antibody levels expressed against IPNV [51]. In terms of antibody responses, IWV vaccines [134] have been linked to high expression levels of IgM and IgT in vaccinated fish [135]. In our studies, we showed a high correlation between postchallenge reduction of mortality and systemic IgM levels, suggesting IgM levels could serve as a correlate of protection for IWV vaccines [81]. Overall, these studies strongly suggest that IWV vaccines are to be considered as exogenous antigens, mainly inducing humoral immune responses.  Table 5 shows the major IWV vaccines explored in fish vaccinology this far.

### 4.2. Subunit Vaccines

The basic principle for this vaccination strategy is that the gene encoding the antigenic proteins is isolated from the native virus and transferred into a heterologous vector that is nonpathogenic for propagation.  Table 6 shows the antigenic proteins identified for the major fish viral diseases and the different expression vectors used for propagation. In the case of VHSV and IHNV, production of subunit vaccines has focused on cloning the G-protein into heterologous vectors [136–138] while, for viruses such as IPNV, the strategy has been to clone the entire outer capsid encoding the protective epitopes, instead of protein segments coding the neutralizing epitopes in heterologous vectors [103, 139–141]. Subunit vaccines are nonreplicative and are delivered exogenously to host cells by the extracellular route (Figure 1). Similar to IWV vaccines, subunit vaccines induce humoral immune responses and upregulation of MHC-II genes [139, 142], which is consistent with observations in higher vertebrates in which it has been shown that immune responses induced by subunit vaccines are mainly dependent on the MHC-II pathway and that they elicit antibody responses [143]. Øvergård et al. [144, 145] showed a high correlation between reduction of viral RNA and activation of CD4 markers in Atlantic halibut (*Hippoglossus hippoglossus* L.) immunized using a subunit vaccine for nodavirus. Although cross presentation of exogenously processed peptides from endosomes into the cytosol has been reported in higher vertebrates [3, 4], there is no study demonstrating cross presentation of peptides processed from endosomes into the cytosol for subunit vaccines in fish.

### 4.3. Virus-Like and Subviral Particle Vaccines

Structural proteins of most viruses self-assemble to forms capsids in different expression systems that resemble the native virus structure in size and morphology and, hence, they are referred to as “virus-like particles” (VLPs). For example, Liu et al. [146] made VLPs of nodavirus expressed in* E. coli* or* Spodoptera frugiperda* (Sf21) insect cells that formed small, nonenveloped *T* = 3 quasi-symmetric particles [147, 148]. They showed that the capsid of* malabaricus* grouper nervous necrosis virus (MGNNV) spontaneously self-assembled into VLPs when expressed in Sf21 cells infected with a recombinant baculovirus [148]. These VLPs were indistinguishable from the native virus particles by electron microscopy and the 3D structure of the VLPs was resolved at 2.3 nm by cryomicroscopy [147]. Similarly, Fang et al. [149] produced VLPs that were devoid of the nucleoprotein but resembled the outer capsid of the native grass carp reovirus (GCRV). However, in some cases VLPs are formed from replication of surface particulate components that do not form the entire capsid, but they contain elements of the outer capsid that are immunogenic. These protein structures are called “subviral particles” (SVPs). Both VLPs and SVPs do not contain the nucleoprotein and as such they are nonreplicative.  Table 7 shows the VLPs, SVPs, and immature virus particles (IVPs) made from different fish viruses. As shown in Table 7, different expression systems were used to make VLPs [139], SVPs [150], and IVPs [151] for IPNV.

Given the similarity to their native viral capsids, VLPs provide an excellent platform for displaying viral epitopes [146, 152]. This property was demonstrated by Lai et al. [153] who produced VLPs for NNV expressed in* E. coli* and showed that NNV failed to infect the Asian sea bass cells that were exposed to the VLPs prior to infection, suggesting that the cell surface receptors were occupied by the VLP-epitopes blocking the wild type virus from entering the cells and thereby protected the cells from developing cytopathic effect (CPE) while control cells not exposed to VLPs developed full CPE. Liu et al. [146] showed that VLPs generated from NNV induced high antibody responses that lasted for more than five months, similar to those produced by the native wild type virus, which were correlated with long-term protection in vaccinated orange spotted grouper. Similarly, Lai et al. [153] produced VLPs in* E. coli* for NNV that expressed high antibody levels, which were correlated with IgM, MHC-II, and CD4 levels in vaccinated fish. These observations suggest VLPs induce the expression of CD4 responses through the MHC-II pathways in a similar pattern to those induced by subunit vaccines in Mammalia [154].

## 5. General Discussion and Conclusion

The most explored strategies for the delivery of antigens using the intracellular route in fish vaccinology involve the use of live and DNA vaccines. The use of DNA vaccines in fish has undergone intense investigation in the last decades as a substitute of replicative antigens for live vaccines. Although factors leading to higher performance of DNA vaccines for rhabdoviruses compared to other fish viral families have not been elucidated, similar observations seen in higher vertebrates show that DNA vaccines for rhabdoviruses are more protective [155, 156] than some of the DNA vaccines for other viral families. In general, replicative vaccines delivered via the intracellular route have been linked to activation of cellular and humoral immune responses in vaccinated fish, which makes these vaccines be more protective than nonreplicative vaccines delivered by the extracellular route.

For antigens administered by the extracellular route, several antigen delivery strategies have been explored in fish vaccinology, which include the use of IWV, subunit, SVP, VLP, and IMP vaccines. In general, all exogenous antigens induce humoral immune responses. In terms of cellular immunity, exogenous antigens were linked to expression of MHC-II and CD4 genes. However, new innovations such as the use of fusion protein and nanoparticles vaccines having the potential to deliver nonreplicative antigens into the cytosol are likely to induce CTL responses in vaccinated fish. The use of nanoparticle vaccines has attracted a lot of interest in the delivery of oral vaccines for fish production systems that require a boost vaccination when fish have been transferred in cages to the sea after prime immunization using parenteral vaccines at the freshwater stage. In general, IWV vaccines are superior to subunit vaccines given that they produce high antibody levels, which correlate with protection in vaccinated fish [81, 157]. In addition, these vaccines have been shown to activate the expression of CD4 and Th2 genes that correlate with high antibody levels consolidating the common notion that exogenous antigens stimulate humoral immune responses orchestrated by Th2 cytokines [154].

Although we did not review the role of APC prime/activated B-cells in antigen uptake and presentation to cells of the adaptive immune system in detail given the limited studies carried out on this topic in fish vaccinology, we can conclude that the different antigen delivery systems explored in fish this far deliver their antigens into the intra- and extracellular compartments and that they activate either the cellular or humoral immune response or both depending on the route of antigen delivery. As pointed out by Howarth and Elliot [158], the most protective vaccines are those that stimulate both the CD4+ and CD8+ T-cells responses and, as such, replicative vaccines such as the live and DNA vaccines that stimulate both the MHC-I and -II pathways are likely to produce better protection in fish (Table 8). So far only the IHNV-DNA vaccine for use in Atlantic salmon in Canada is the only one licensed while live viral vaccines are feared to revert to virulence. Hence, the use of IWV vaccines which accounts for the largest proportion of licensed vaccines is likely to continue dominating the vaccine industry in aquaculture [81, 157]. Therefore, the search for better antigen delivery systems that stimulate both CD4+ and CD8+ responses that have the potential to induce long-lasting protective immunity has to continue. Overall, we anticipate that the synopsis of different antigen delivery systems presented here will shed new insights into the limitations and successes of the current immunization strategies used in fish vaccinology.

## Figures and Tables

**Table 1:** Fish antigen presenting and adaptive immunity cell receptors.

Protein	Selected examples of fish species	Reference
(1) Antigen presenting surface markers and MHC molecules		
CD80 (B7.1)	Zebrafish, rainbow trout	[44, 159]
CD83 (7.2)	Zebrafish, turbot, Atlantic salmon	[44, 159–161]
CD86	Zebrafish, rainbow trout	[44, 159]
CD209	Zebrafish	[162]
MHC-I	Orange spotted grouper, sea bass, grass carp	[49, 163, 164]
MHC-II	Zebrafish, lake trout,	[159, 165]

(2) T-cell receptors, costimulatory and activator molecules		
CD3*γ*	Atlantic salmon	[166]
CD3*ε*	Atlantic salmon	[166]
CD3*ζ*	Atlantic salmon	[166, 167]
CD3*δ*	Rainbow trout, Atlantic salmon	[166, 168, 169]
TCR*α*	Rainbow trout and Japanese flounder	[170, 171]
TCR*β*	Rainbow trout and Japanese flounder	[170, 171]
TCRΥ	Japanese flounder	[171]
TCR*σ*	Japanese flounder	[171]
CD28	Rainbow trout	[47, 48]
CTLA	Rainbow trout	[47, 48]
CD40L	Atlantic salmon and Japanese flounder	[65, 172]

(3) T-cells		
CD8*α*	Rainbow trout, Atlantic salmon	[51, 143, 173]
CD8*β*	Atlantic salmon, Atlantic salmon	[143, 169]
CD4	Atlantic salmon	[169]

(4) Immunoglobulins		
IgM	Atlantic salmon, rainbow trout	[56, 174, 175]
IgD	Atlantic salmon, rainbow trout	[59, 174]
IgT	Rainbow trout, Atlantic salmon	[58, 62]
IgZ	Zebrafish	[176]

**Table 2:** Live vaccines.

Virus	Abbreviation	Fish host	Mode of attenuation	Protection	Reference
Cyprinid herpesvirus subtype 3	CyHV-3	Carp	Natural selection	High	[177]

Viral hemorrhagic septicemia	VHSV	Rainbow trout	Naturally attenuated	High	[178]
	VHSV	Rainbow trout	Naturally attenuated	High	[84]
	VHSV	Olive flounder	Recombinant (RG) modification	High	[179]
	VHSV	Rainbow trout	Recombinant (RG) modification	High	[180]
	VHSV	Zebra fish	Recombinant (RG) modification	High	[181]

Infectious hematopoietic necrosis virus	IHNV	Rainbow trout	Multiple serial passage	High	[86]
	IHNV	Rainbow trout	Naturally attenuated	High	[83]
	IHNV	Rainbow trout	Natural selection	High	[182]
	IHNV	Rainbow trout	Recombinant (RG) modification	High	[91]
	IHNV	Rainbow trout	Recombinant (RG) modification	High	[183]

Infectious pancreatic necrosis virus	IPNV	Atlantic salmon	Avirulent strain/low dose	High	[82]

Rock bream iridovirus	RSIV	Rock bream	Low temperature	High	[184]

**Table 3:** DNA vaccines explored in fish.

Classification	Virus family	Pathogen	Abbreviation	Antigen	Protection^∗^	Reference
DNA viruses	Iridovirus	Red sea bream iridovirus	RSIV	Major capsid	Moderate	[185]
	Herpesviridae	Channel catfish virus (CCV)	CCV	ORF 6&59	Low	[186]

RNA virus	Rhabdoviridae	Viral hemorrhagic septicemia virus	VHSV	G	High	[95]
	Rhabdoviridae	Infectious hematopoietic necrosis virus	IHNV	G	High	[98, 187, 188]
	Rhabdoviridae	Spring viremia of carp virus	SVCV	G	High	[189, 190]
	Rhabdoviridae	*Hirame rhabdovirus *	HRV	G	High	[191]
	Birnaviridae	Infectious pancreatic necrosis virus	IPNV	SegA/VP2	Moderate	[103, 140]
	Orthomyxoviridae	Infectious salmon anemia virus	ISAV	HE	Moderate	[192]
	Togaviridae	Salmon alphavirus subtype 3	SAV-3	E2	Moderate	[157]
	Nodaviridae	Atlantic halibut nodavirus	ANHV	Capsid	Low	[193]

^∗^Protection was determined by postchallenge relative percent survival (RPS).

**Table 4:** Nanoparticle vaccines.

Virus	Virus	Fish host	Admin	Antigen	Type	Protection^∗^	Reference
Lymphocytic virus	LCDV	Japanese flounder	Oral	Plasmid DNA	PLGA	High	[194]
Infectious hematopoietic necrosis virus	IHNV	Rainbow trout	Oral	Plasmid DNA	PLGA	Low	[195]
Infectious pancreatic necrosis virus	IPNV	Atlantic salmon	Injection	IWV	PLGA	Low	[127]
Infectious pancreatic necrosis virus	IPNV	Atlantic salmon	Injection	IWV	PLGA	Low	[103]
White syndromes spot virus	WSSV	Shrimp	Oral	Plasmid DNA	Chitosan	ND	[112]
White syndromes spot virus	WSSV	Shrimp	Oral	Plasmid DNA	Chitosan	ND	[196]

^∗^Protection was determined by postchallenge relative percent survival (RPS). ND = not done (not tested for protection).

**Table 5:** Inactivated whole virus vaccines explored in fish.

Pathogen	Abbreviation	Virus family	Fish species	Protection	Reference
Viral hemorrhagic septicemia virus	VHSV	Rhabdoviridae	Rainbow trout	High^∗^	[197]
Infectious hematopoietic necrosis virus	IHNV	Rhabdoviridae	Rainbow trout	High^∗^	[198]
Spring viremia of carp virus	SVCV	Rhabdoviridae	Carp	High^∗^	[199]
Infectious pancreatic necrosis virus	IPNV	Birnaviridae	Atlantic salmon	High^∗^	[81, 103]
Salmon pancreas disease virus	SPDV	Togaviridae	Rainbow trout	High^2^	[200]
Red seabream iridovirus	RSIV	Iridovirus	Sea bass	High^∗^	[201]
Singapore grouper iridovirus	SGIV	Iridovirus	Grouper	High^∗^	[202]
Channel catfish virus	CCV	Herpesviridae	Catfish	Moderate/high^∗^	[203]
Cyprinid herpesvirus subtype-3	CyHV-3	Herpesviridae	Carp	High^∗^	[204]
Nervous necrosis virus	NNV	Betanodaviridae	Grouper	High^1^	[205]
Nervous necrosis virus	NNV	Betanodaviridae	Sea bass	High^1^	[206]
Salmon anemia virus	SAV	Orthomyxoviridae	Atlantic salmon	High^∗^	[207]

Protection measured by ^∗^relative percent survival (RPS), ^1^protection against postchallenge virus infection, and ^2^pathology.

**Table 6:** Subunit vaccines.

Virus	Abbreviation	Protein	Vector	Efficacy	Fish species	Reference
Infectious pancreatic necrosis virus	IPNV	VP2	*Escherichia coli *	Low	Atlantic salmon	[103]
	IPNV	VP2	Yeast cells	Low	Rainbow trout	[139]
	IPNV	VP2/3	*Lactobacillus casei *	Low	Rainbow trout	[208]
	IPNV	VP2	Baculovirus	Low	Atlantic salmon	[141]
	IPNV	VP2	Semliki Forest virus	N/A	CHSE cells	[209]

Viral hemorrhagic septicemia virus	VHSV	G	*Saccharomyces cerevisiae *	—	Rainbow trout	[210]
	VHSV	G	*Escherichia coli *	High	Rainbow trout	[197]
	VHSV	G	*Yersinia ruckeri *	Moderate-high	Rainbow trout	[211]
	VHSV	G	Baculovirus	high	Rainbow trout	[142]
	VHSV	G	*Edwardsiella tarda *	Moderate	Olive flounder	[212]

Infectious hematopoietic necrosis virus	IHNV	G	*Escherichia coli *	Moderate	Rainbow trout	[138]
	IHNV	G	*Caulobacter crescentus *	Low	Rainbow trout	[213]
	IHNV	G	Baculovirus	Moderate	Rainbow trout	[214]
	IHNV	G	*Aeromonas salmonicida *	Moderate-high	Rainbow trout	[137]

	GCRV	VP4	*Escherichia coli *	Moderate-high	Grass carp	[215]
	GCRV	VP5, VP7	*Escherichia coli *	Moderate	Grass carp	[216]

	VER	Capsid	*Escherichia coli *	Low	Atlantic halibut	[217]

**Table 7:** Subviral, immature, and virus-like particles used for fish vaccines.

Virus	Classification	Protein	Cells/vector	Fish host	Protection	Reference
NNV	VLP	Capsid	*Escherichia coli *	Orange spotted grouper	ND^∗^	[153]
NNV	VLP	Capsid	*Saccharomyces cerevisiae *	Red spotted grouper	ND^∗^	[218]
IPNV	VLP	VP2	Baculovirus/insect larvae	Rainbow trout	Low	[219]
IPNV	IVP	VP2	CHSE cells	Rainbow trout	ND^∗^	[151]
IPNV	SVP	VP2	Yeast cells	Rainbow trout	Low	[150]
IPNV	SVP	VP2	Yeast cells	Rainbow trout	Low	[139]
GCRV	SVP	Capsid	*Ctenopharyngodon idellus *	Grass carp	ND^∗^	[149, 220]
VNNV	VLP	Capsid	Baculovirus	European sea bass	High	[221]
NNV	VLP	Capsid	Baculovirus	Orange spotted grouper	High	[148]
NNV	VLP	Capsid	*Escherichia coli *	Dragon and Malabar grouper	ND^∗^	[146]
VHSV	Peptide	Nucleoprotein		Rainbow trout	ND^∗^	[136]

ND = Note done (No protection studies carried out).

^∗^Only immune expression studies were carried out by enzyme linked immunosorbent assay (ELISA) or gene expression.

**Table 8:** Comparison of the intra- and extracellular antigen processing parameters.

Parameters	Intracellular antigen delivery	Extracellular antigen delivery
Vaccine types		
Viability of antigens	Mostly replicative	Nonreplicative
Examples of vaccine types	Live and DNA vaccines	IWV vaccines, subunit vaccines
Antigen uptake and presentation		
Pathway of uptake into host cells	Endogenous pathway	Exogenous pathway
Antigen uptake and processing	Penetration of host cell membrane	Phagocytosis by APCs
Site of antigen deposition	Cytoplasm	Endosome/phagosome
Antigen presenting molecules	MHC-I and MHC-II	MHC-II
Mode of antigen processing	Proteosomal degradation	Endosomal degradation
Adaptive immunity		
Type of immune response induced	Cellular and humoral immune responses	Humoral immune responses
Cell types involved	B- and T-lymphocytes	B-lymphocytes
T-cell subtypes	CD4 and CD8 T-cells	CD4 T-cells
Effector molecules/cells	CTL (cellular) and antibodies (humoral)	Antibodies
Effector mechanisms	CTL killing of virus infected cells	Antibody-neutralization of virus
	Antibody-neutralization of virus	
